# CONFLICT MONITORING AND INHIBITORY CONTROL IN OLDER ADULTS WITH AGE-RELATED HEARING LOSS

**DOI:** 10.1093/geroni/igae098.0403

**Published:** 2024-12-31

**Authors:** Shraddha Shende, Raksha Mudar

**Affiliations:** Illinois State University, Normal, Illinois, United States; University of Illinois, Champaign, Illinois, United States

## Abstract

Various processes of cognitive control, including conflict monitoring and inhibitory control, are critical for listening in complex environments (e.g., speech-in-noise [SiN] recognition). However, few have examined both conflict monitoring and inhibitory control in individuals with age-related hearing loss (ARHL) despite their known challenges in SiN recognition. In 16 individuals with mild ARHL and 23 normal-hearing controls, we examined conflict monitoring using two picture-word interference tasks, “Animals” and “Objects” tasks; inhibitory control in two Go/NoGo tasks, “Single-Car” and “Object-Animal” task; and SiN using QuickSiN test. The picture-word interference tasks consisted of pictures of animals/objects that were superimposed with labels that either matched (matched condition) or did not match the pictures (unmatched condition). Participants responded by pushing the “matched” or “unmatched” button. Reaction time and accuracy were recorded. The Go/NoGo tasks consisted of pictures of cars/dogs in the Single-Car task and objects/animals in the Object-Animal task. Participants responded by pushing a button to some trials, the Go trials (cars/objects), and withholding button push to others, NoGo trials (dog/animals). Reaction time for Go trials and accuracy for Go/NoGo trials were recorded. Relative to controls, ARHL group had worse accuracy on unmatched condition of “Objects” task (p =.006) and Go trials of “Object-Animal” task (p =.018). Partial correlations revealed that worse SiN recognition was associated with worse accuracy on Go trials of the “Object-Animal” task, and no other correlations were significant. Our findings reveal alterations in both conflict monitoring and inhibitory control in mild ARHL, with inhibitory control linked to SiN recognition.

